# Validity and reliability of new instruments for measuring patient satisfaction with removable dentures, Arabic Version

**DOI:** 10.1186/s12903-021-01811-w

**Published:** 2021-09-15

**Authors:** Ahmad Al Jaghsi, Musab Saeed, Salem Abu Fanas, Ahmed Yaseen Alqutaibi, Torsten Mundt

**Affiliations:** 1 College of Dentistry, Restorative Department, Clinical Sciences Department, Ajman University, University Street, Al jerf 1, Ajman, United Arab Emirates; 2 Center of Medical and Bio-Allied Health Sciences Research, Ajman University, Ajman, United Arab Emirates; 3 Department of Prosthodontics, Gerodontology and Dental Materials, Greifswald University Medicine, Greifswald, Germany; 4 College of Dentistry, Department of Prosthodontics, Taibah University, Medina, Saudi Arabia

**Keywords:** Questionnaire, Index, Patient satisfaction, Removable denture, Validity, Reliability

## Abstract

**Background:**

The psychometric properties of self-administered instruments for measuring patient satisfaction with removable dentures should be tested before inviting patients to express their opinions. This study aimed to evaluate the validity and reliability of new instruments in the Arabic language that measure patient satisfaction with all types of removable dentures.

**Methods:**

A three-step methodology was used to translate and test the instruments. In step one, the instruments were translated from tested German instruments to develop the pilot questionnaires. In step two, the face validity of the pilot questionnaires was tested through three rounds of interviews. There were 15, 13, and 15 participants per round, respectively. At the end of every round, the results of the interviews were discussed with an expert panel. The expert panel confirmed the form and the type of questionnaires’ adjustments before a new round of interviews began. At the end of step two, the final form of the questionnaires was reached. In step three, 235 questionnaires were distributed to 133 participants to estimate the construct validity of the upper jaw and the lower jaw questionnaires. After one week, the participants were asked to complete the questionnaires again. A total of 102 questionnaires were returned and used to assess the instruments’ reliability. Factor analysis was used to assess the construct validity. The intraclass correlation coefficient and Cronbach’s alpha were used to estimate the reliability and suitability of the items in the indexes.

**Results:**

The result of step one was two pilot questionnaires. The pilot questionnaires were adjusted in step two. At the end of step two, the questionnaires proved to have good face validity. Factor analyses in step three revealed that only one factor could be retained. The one-factor model explained 60.95% and 63.06 of the total variance of the upper jaw and lower jaw questionnaires, respectively. The items in every questionnaire shared the same cluster and could be summed to form an upper jaw index and lower jaw index that reflected patient satisfaction with removable dentures. Cronbach’s alpha values indicated excellent internal consistency and reliability for the upper jaw questionnaire (α = 0.91) and the lower jaw questionnaire (α = 0.92). Intraclass correlation coefficient values ranged from 0.72 to 0.95, which can be considered “moderate” to “excellent”.

**Conclusions:**

The Arabic version of questionnaires and indexes assessing patient satisfaction with upper and lower removable dentures are reliable and valid self-administered instruments.

**Supplementary Information:**

The online version contains supplementary material available at 10.1186/s12903-021-01811-w.

## Introduction

Patient satisfaction has gained increasing attention from dental health providers and the medical industry. Consequently, different methods and strategies have been developed and implemented to measure and improve patient/customer satisfaction. Nevertheless, evidence shows that more work in this field is still needed [1]. Patient satisfaction can be estimated effectively through two important methods: interviews and questionnaires [2].

By asking patients the right questions, there is almost limitless and useful information that can be collected. In some cases, relying on observation or clinical examination to collect the needed information is not a practical approach. Instead, it is preferable to ask a valid and reliable set of questions. A questionnaire is a type of communication medium between the participants and the researcher [3]. The role of a questionnaire is to provide a standardized approach across all participants [3]. In self-administered questionnaires, questions are asked in the same way and in a format that both matches the needs of the study and is convenient for participants. If these conditions are not met, it will be challenging for the researcher to interpret the answers.

First and foremost, survey instruments should have special psychometric properties when they are self-administered. Annie G. defined the psychometric properties of an instrument as “the construction and validation of the measurement instrument” [4]. These properties reflect the instrument’s reliability and validity [5]. Patient satisfaction is a subjective assessment [6]. Therefore, the psychometric properties of the instrument should be tested before inviting patients to express their opinions. If the study fails to satisfy this necessity, the collected data will be questionable, and the survey conclusion cannot be trusted [7]. That is, testing the validity and reliability of the questionnaire helps the researcher estimate the level of accuracy and consistency of the collected data. Under these two components, there are several subtypes: face validity and construct validity, on the one hand, and reliability and internal consistency, on the other hand.

Face validity may best be understood as reflecting the extent to which the instrument measures what it is intended to measure [8]. Face validity, which is a form of content validity, plays an essential role in constructing and testing the questionnaire [9]. This type of validity can be tested through expert assessment of items and interviews [10, 11].

Construct validity reflects how far the questionnaire or the index truly evaluates the hypothesis, theory or themes under examination. It should reveal that the ‘items’ (questions) scores on a specific domain anticipate the theoretical trait it claims to predict [12]. One of the good and most widely used methods to estimate construct validity is confirmatory factor analysis [13].

Instrument reliability means that the instrument consistently reflects the construct that it is measuring by giving the same score if used over time or across multiple administrations. The stability of scores over time requires that all other things be equal [14]. This means that in our study, no adjustment or changes occurred intraorally or to the removable dentures (RDs) between the first and second assessments. Intraclass correlation coefficients (ICCs) are an important element for test–retest reliability assessment [15].

Internal consistency, as described by Revelle, is the degree to which all items quantify the same construct [16]. One of the widely used methods for estimating the internal consistency of a questionnaire or index is Cronbach’s alpha. It helps quantify the reliability of a score and summarize the data from multiple questionnaire items [17].

Although patient satisfaction is an important subject, we could not find in the dental literature a valid and reliable instrument in the Arabic language that measures patient satisfaction with RDs.

The aim of this study was to evaluate the validity and reliability of new instruments in the Arabic language that measure patient satisfaction with all types of RDs. The Arabic version was translated from valid and reliable German instruments [7].

## Method and materials

The study is in full accordance with the World Medical Association Declaration of Helsinki. The medical ethics committee at Ajman University approved the study. All participants gave their written informed consent to all study procedures.

The participants included in the study were Arabic-speaking patients who had a partial or complete RD. RDs can be tissue-, teeth-, implant- or combined-supported dentures. The participant should be adapted and wearing their RDs for at least two months. If patients had intra- or extraoral pathological findings, were not wearing their RDs regularly or were classified in one of the ten main groups of the international classification of diseases or mental and behavioral disorders [18], they were excluded from the study. All the patients included in the study were treated at Ajman University between 2015 and 2019.

**Fig. 1:**
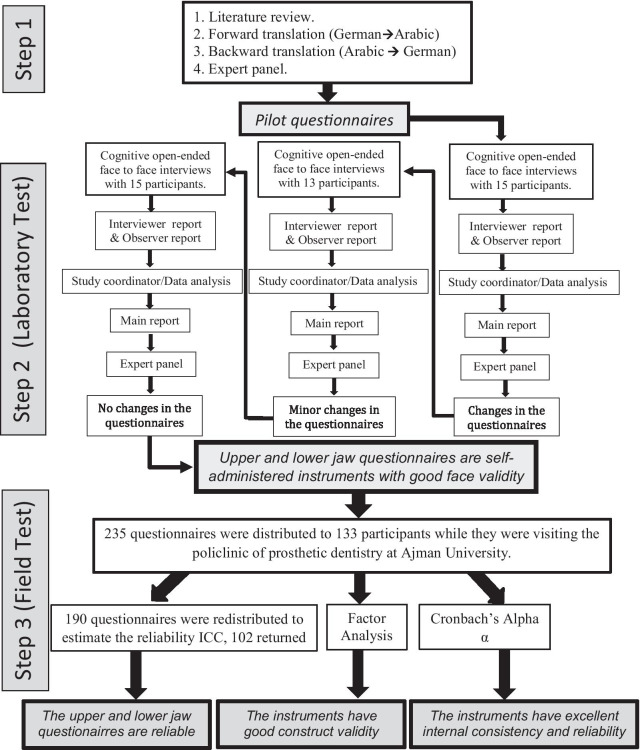
Study design

The study went in three steps (Fig. 1).

### Step one

Two bilingual translators (one was a prosthodontist) translated the eight-item German questionnaire for the upper jaw and the second eight-item questionnaire for the lower jaw to the Arabic language (forward translation). The Arabic version was translated back (backward translation) to German by two new bilingual translators (one was a prosthodontist). In accordance with Guillemin et al.’s recommendation, only one in every couple was aware of the questionnaire’s intended concepts [19]. This helps in detecting subtle differences with the original questionnaire. Any discrepancies, misunderstandings or unclear wording at the end of the forward or backward translation landed in the hands of the expert panel. The expert panel reviewed the translation, resolved the discrepancies and finalized the Arabic version.

A 5-point Likert scale was used to capture the participants’ responses for every item (question). The scale range was very good (1), good (2), neither good nor bad (3), bad (4) and very bad (5). The sum of all items represents the satisfaction index with the RD; the higher the index value, the less satisfied the participant was. At the end of this step, two pilot questionnaires for measuring patient satisfaction with their upper and lower RDs were constructed.

### Step two (laboratory test)

In steps two and three, we follow the methodology used to develop the German instruments [7].

For the second step, two dentists were trained to conduct cognitive interviews: one as the interviewer and the second as the observer. They reported the interview results separately to the study coordinator.

Step two aimed to test the face validity of the questionnaires. Three rounds were sufficient to test the face validity using the cognitive interviewing approach with 43 participants. The approach is described in a previous paper [7]. The interviewer read the questions to the participants in the first round. In the second and third rounds, he handed the questionnaires to the participants so they could read aloud and answer the items. During the interview, the interviewer tried to assess the degree to which the questionnaire subjectively measured the domains that it was supposed to be measured. He assessed whether the items were clear and whether the patient answered what truly needed to be answered. He also tried to estimate the appropriateness and suitability of the items. To achieve that, two methods were used: think aloud and verbal probing. The most commonly used probes were overt encouragement, silent probe, repetition, comprehension, elaboration, asking for clarification and paraphrasing. The observer primarily reported the interview quality. He described whether the interviewer was able to extract the needed information without misusing the probes or using leading statements. He also described the interaction between the interviewer and the participants and how seriously the participants engaged in the interview. At the end of every round, the interviewer and observer reports were handed to the study coordinator. The reports were subjected to data analyses to form the main report. Three main reports were made. The main report was discussed with the expert panel. All members of the expert panel were aware of the study methodology and the construct of interest. Two of them were bilingual, and one was a developer of the original German questionnaires. They reviewed the interviews’ results (the main report) and confirmed the needed adjustments in the pilot questionnaires, which were suggested by the interviewer, observer and study coordinator. Subsequently, the form and the type of questionnaire adjustments were decided before a new round of interviews was begun. The adjustments in the questionnaires aimed mainly at improving face validity and participants’ acceptance of the instruments.

### Step three (field test)

This step aimed at estimating the construct validity and reliability of the upper jaw and the lower jaw satisfaction questionnaires and test the two indexes’ validity and reliability.

To achieve that, 235 questionnaires were distributed to 133 participants. After one week, the participants were asked to complete the questionnaires again.

Factor analysis was used to assess the construct validity and to detect the factors that underlie the study dataset. Factor analysis was also used to construct the two indexes. The intraclass correlation coefficient (ICC) and Cronbach’s alpha were used to estimate the reliability and suitability of the items in the index. A p-value below 0.05 was considered statistically significant. The statistical analysis was performed using IBM® SPSS 25.0 (SPSS Inc).

## Results

### Step one

The result of the two-way translation of the tested German version was two pilot questionnaires, one for the upper jaw and the second one for the lower jaw. Every questionnaire contained eight items. The first item concerned patient satisfaction with RD in general. The other seven items cover the following domains: eating, speaking, esthetics, cleanability of the removable partial denture (RPD) and RPD retention, support and stability.

### Step two (laboratory test)

Fifteen participants (8 males) engaged in the first round of interviews. The participants were wearing 21 RDs: six CDs, seven RPDs with metal framework, and two acrylic RPDs. Ten of the 21 RDs were in the upper jaw.

The interview duration ranged from 15 to 25 min. At the end of every interview, the interviewer and the observer reports were subjected to data analyses (Table 1). Subsequently, the main report, which was discussed with the expert panel, was written. After the first round of interviews, one of the 15 participants faced minor difficulties in understanding what we truly needed him to understand in items two, three, and four. Adjustments in the items, which measured patient satisfaction with RD retention, support, and stability, were made by adding elaboration.

**Table 1:** Data analysis of the interviewer and observer reports

Steps of data analysis
1. Settling the key questions which should be answered at the end of the analyses: Assess clarity, patients' comprehension, and understanding of the items Assess the appropriateness and suitability (readability and layout design) of the questionnaires Assess acceptability
2. Determining the quality of the data (observer report is a cornerstone in this step)
3. Identifying the ability to answer the previous points

The second round of interviews included 13 participants (7 males). Nine of the 18 RDs were in the upper jaw. The participants were wearing 5 CDs (upper and lower), seven RPDs with metal framework, and one acrylic RPD. Interviews revealed that all participants were able to understand the items. Data were analyzed, and the main report was formulated. The expert panel held a meeting to discuss the results of the interviews, and a decision was made to make no additional major changes, except for bolding the items’ keywords to increase their readability.

The last round of interviews included 15 participants (7 males) wearing 21 RDs. The participants were wearing 6 CDs (upper and lower), seven RPDs with a metal framework and two acrylic RPDs. Twelve of the 21 RDs were in the upper jaw. The second- and third-round confirmed that all the participants comprehended all items, and both questionnaires were self-administered instruments with good face validity.

### Step three (field test)

Two hundred thirty-five questionnaires were distributed to 133 participants with a mean age of 65. At the time of examination, the mean usage of the RDs was 21.5 months (Table 2).

**Table 2:** Descriptive statistics for the dataset of the study third step

	Upper Jaw	Lower Jaw	Male	Female	Complete denture	RPD with metal framework	Acrylic RPD
Frequency (N)	109	126	70	63	109	103	23
Percent (%)	46.4	53.6	52.6	47.4	46.4	43.8	9.8
Total	235		133		235		

The correlation matrix between the items (Table 3), Bartlett’s sphericity test and Kaiser–Meyer–Olkin test (KMO) were used to estimate whether the dataset was suitable for factor analysis. The correlation matrix between the items was acceptable, not very high or very low, with a determinant equal to 0.005. Bartlett’s test results were statistically significant. The KMO results for the upper jaw and lower jaw questionnaires were 0.87, which is considered meritorious according to some researchers [20]. Therefore, it was confirmed that the dataset is suitable for factor analyses.

**Table 3:** Correlation matrix between the items

	Items of the upper jaw questionnaire, N = 109									Items of the lower jaw questionnaire, N = 126							
	I^a^1	I2	I3	I4	I5	I6	I7	I8		I1	I2	I3	I4	I5	I6	I7	I8
I1	1.00	0.71	0.67	0.57	0.42	0.75	0.42	0.46	I2	1.00	0.62	0.68	0.62	0.60	0.63	0.40	0.58
I2	0.71	1.00	0.85	0.67	0.63	0.69	0.48	0.40	I3	0.62	1.00	0.68	0.49	0.49	0.76	0.32	0.42
I3	0.67	0.85	1.00	0.62	0.57	0.68	0.46	0.33	I4	0.68	0.68	1.00	0.72	0.63	0.68	0.61	0.58
I4	0.57	0.67	0.62	1.00	0.56	0.63	0.33	0.62	I5	0.62	0.49	0.72	1.00	0.64	0.64	0.55	0.67
I5	0.42	0.63	0.57	0.56	1.00	0.50	0.41	0.36	I6	0.60	0.49	0.63	0.64	1.00	0.50	0.45	0.55
I6	0.75	0.69	0.68	0.63	0.50	1.00	0.59	0.54	I7	0.63	0.76	0.68	0.64	0.50	1.00	0.51	0.56
I7	0.42	0.48	0.46	0.33	0.41	0.59	1.00	0.33	I8	0.40	0.32	0.61	0.55	0.45	0.51	1.00	0.48
I8	0.46	0.40	0.33	0.62	0.36	0.54	0.33	1.00	I1	0.58	0.42	0.58	0.67	0.55	0.56	0.48	1.00
Determinant = 0.005									Determinant = 0.005								

^a^I is Item

Eight linear components were identified for every questionnaire (Table 4). The analysis of the dataset revealed that for every questionnaire, only one factor could be retained. The retained factor had an eigenvector over 1.0 (Table 4). The factor explains 60.95% and 63.06 of the upper jaw and lower jaw questionnaires’ total variance, respectively. The component matrix shows that the items’ loadings onto the extracted factor were high, > 0.6 (Table 5). Therefore, the one-factor model was reliable [21, 22]. Subsequently, the items’ scores could be summed to form an upper jaw index and a lower jaw index for measuring patient satisfaction with their RD [22, 23].

**Table 4:** Factor extraction from the upper jaw questionnaire and the lower jaw questionnaire

Items of the upper jaw questionnaire, N = 109				Items of the lower jaw questionnaire, N = 126			
Factor	Initial eigenvalues			Factor	Initial eigenvalues		
	Total	% of Variance	Cumulative %		Total	% of Variance	Cumulative %
1	4.88	60.95	60.95	1	5.04	63.06	63.06
2	0.83	10.39	71.34	2	0.81	10.07	73.13
3	0.73	9.08	80.42	3	0.61	7.62	80.75
4	0.63	7.82	88.24	4	0.48	5.94	86.70
5	0.33	4.15	92.39	5	0.34	4.29	90.99
6	0.26	3.22	95.61	6	0.31	3.89	94.88
7	0.21	2.60	98.21	7	0.26	3.24	98.12
8	0.14	1.79	100.00	8	0.15	1.88	100.00

Extraction Method: Principal Component Analysis

**Table 5:** Factor 1 and item's loading

Upper jaw questionnaire		Lower jaw questionnaire	
Item	Loading	Item	Loading
I1	0.81	I1	0.81
I2	0.89	I2	0.76
I3	0.85	I3	0.88
I4	0.81	I4	0.85
I5	0.71	I5	0.77
I6	0.87	I6	0.83
I7	0.63	I7	0.67
I8	0.63	I8	0.76

Cronbach’s alpha (α) was used to assess the internal consistency of the questionnaires [22]. Cronbach’s alpha for the upper jaw questionnaire was α = 0.91. For the lower jaw questionnaire, it was α = 0.92 (Table 6). This reflects excellent instruments internal consistency [24].

**Table 6:** Cronbach's Alpha; upper jaw questionnaire and lower jaw questionnaire

Items	Upper jaw questionnaire, N = 109			Lower jaw questionnaire, N = 126		
	Corrected Item-Total Correlation	Squared Multiple Correlation	Cronbach's Alpha if Item Deleted	Corrected Item-Total Correlation	Squared Multiple Correlation	Cronbach's Alpha if Item Deleted
I1	0.74	0.64	0.89	0.75	0.59	0.90
I2	0.83	0.79	0.88	0.68	0.68	0.91
I3	0.78	0.75	0.89	0.84	0.73	0.89
I4	0.74	0.64	0.89	0.78	0.67	0.90
I5	0.63	0.46	0.90	0.69	0.52	0.91
I6	0.82	0.72	0.88	0.78	0.71	0.90
I7	0.54	0.39	0.91	0.59	0.47	0.91
I8	0.54	0.46	0.91	0.68	0.52	0.91
Cronbach’s Alpha	0.91			0.92		

Eighty-seven participants (47 male, 54%) wearing 102 RDs (52 upper jaws) completed the questionnaires again. The RDs were nine acrylic RDs, 44 RDs with metal framework and 49 complete dentures. The participants answered the second questionnaire one week after filling out the first questionnaire. The ICC (test–retest) values ranged from 0.72 to 0.95 (Table 7). Therefore, the level of reliability can be considered “moderate” to “excellent”. [25]

**Table 7:** Intra-class correlation coefficient (test–retest)

Item	Upper jaw questionnaire n = 52	Lower jaw questionnaire n = 50
1	0.89 (0.81–0.93)^a^	0.83 (0.72–0.90)
2	0.91 (0.85–0.95)	0.90 (0.83–0.94)
3	0.92 (0.87–0.96)	0.88 (0.80–0.93)
4	0.93 (0.89–0.96)	0.89 (0.82–0.94)
5	0.91 (0.85–0.95)	0.84 (0.74–0.91)
6	0.94 (0.90–0.97)	0.91 (0.85–0.95)
7	0.88 (0.80–0.93)	0.87 (0.78–0.92)
8	0.90 (0.83–0.94)	0.90 (0.83–0.94)
Index	0.92 (0.86–0.95)	0.84 (0.74–0.91)

^a^At 95% CI (lower bound, upper bound)

## Discussion

The Arabic version of the upper jaw questionnaire and the lower jaw questionnaire to measure patient satisfaction with RDs proved reliable and valid. Factor analysis identified a single factor with an eigenvalue above 1. The items in every questionnaire shared the same cluster and could be summed to form an upper jaw index and lower jaw index that reflected the level of patient satisfaction with their RDs. This cluster contained eight items covering the following domains: speaking, eating, appearance, cleanability, RPD movement in three directions (retention, stability and support) and overall patient satisfaction. The two indexes proved to be reliable with excellent internal consistency.

Quality control and quality assurance processes were applied throughout the study steps. The first level was the interview observer. The second level was the study coordinator, who compared the interviewer report with the observer report. He documented any discrepancy between the reports before performing the data analyses and drafting the main report. The third level was the expert panel, which continually monitored the study until the end by holding regular meetings with the investigators to track progress and assess how much the investigators adhered to the given study guidelines. This layering of control helps improve the quality of the study and strengthen the confidence in the study conclusion.

However, the study had several limitations. It was not a multicenter study, and all data were collected from patients treated at Ajman University. COVID-19-related shutdowns reduced the number of participants and interrupted the study flow. The authors discarded the questionnaires that were returned from the participants who were asked to complete the questionnaires again if they sent it after 5 weeks of completing the first questionnaire.

At the end of the second round of interviews, all 13 participants expressed a full understanding of the items. According to previous studies [26, 27], to reach data saturation in qualitative studies, 12 is considered the minimum sample size. Subsequently, 13 can be viewed as sufficient for the data analysis. Nevertheless, a third round with 15 participants was performed to emphasize the results that both questionnaires were self-administered instruments with good face validity. No additional interviews after the third round were conducted because the participants stopped adding new contributions to the existing findings and holding more interviews was considered repetitious of comments and themes [28].

The plan was to collect 300 questionnaires. Unfortunately, the COVID-19-related shutdowns forced the researcher to close the study at a sample size of 235. A sample size of 200 should be considered fair [29]. Moreover, some researchers believe that a sample size between 100 and 200 can be good enough if there are communalities after extraction in the 0.5 range [30]. Others stated that a sample size of 100 is not recommended, unless the loading is greater than 0.51 [30, 31]. In the current study, all items had a loading ≥ 6 (Table 5). Therefore, the study sample size can be considered acceptable.

Many studies have been conducted to determine the different aspects of patient satisfaction with RDs. These aspects can be categorized into two groups. The first group is RPD-related aspects, such as fit, chewing, speech, appearance, denture cleanliness, occluding teeth, distribution of chewing forces, type of RPD and RPD location (upper or lower jaw) [32–36]. The second is patient-related aspects, such as the patient’s personality, socioeconomic status, emotional status, expectations, patient-doctor relationship, prior experience with RPDs, age, sex, natural tooth problems, oral cavity status and general health status [36–39]. Although the importance of the previous aspects cannot be denied, not all of them were covered by the study questionnaires. Only the aspects in the first group were considered in the study questionnaires. The other aspects are confounders or not directly related to patient satisfaction with RDs. Some aspects require more extended and time-consuming instruments to be measured. However, we aimed to develop a relatively quick-to-complete questionnaire that was easy to understand and walk through. Moreover, we tried to make the questionnaire more suitable for elderly individuals, who represent the majority of RD wearers. [40] Accordingly, the 5-point Likert scale can be considered a good choice because it supports the questionnaire’s simplicity. Additionally, it is the scale used in many well-known instruments, such as the OHIP-14 or OHIP-EDENT.

Factor loadings help estimate the relative contribution that an item makes to a factor and represent the correlation of each original item with the selected factor. A loading greater than 0.3 is considered necessary by some researchers [22]. Others consider 0.4 the cutoff point [31]. All the items in the two questionnaires had a loading of ≥ 0.6 (Table 5). Therefore, the factor can be considered reliable [21], and all items are of substantive importance to the one-factor model, and none of them should be ignored [22]. This underlying one dimension (factor) reflects constructs that cannot be measured directly, which is patient satisfaction with their RD. However, this factor can be estimated by summing the eight items to form a patient satisfaction dimension that creates satisfaction with the upper jaw RD index and satisfaction with the lower jaw RD index.

The ICC was used to compare participants’ estimation of their satisfaction with RD at different times. The assessment of the questionnaire or index reliability was conducted at the item level and the index level. For ICC calculation, we considered the index scores and the order of item scores as well as their relative values (absolute agreement). The data were analyzed using a single measurement. The analyses showed that the upper jaw questionnaire and index test–retest reliability was “good” to “excellent”, and the lower jaw questionnaire and index test–retest reliability was “moderate” to “excellent”.

Cronbach’s alpha was used to measure the instruments’ internal consistency and scale reliability and to determine the degree to which all the questionnaire items measured the same construct. Cronbach’s alpha was 0.91 and 0.92 for the upper jaw and lower jaw, respectively. These values indicated that the scale was reliable. Table 6 shows that we attempted to improve the reliability (increasing the α value) by removing the unfitted item from the index. The results showed that all the items fit in their instrument, and none should be removed to raise the α value above 0.91 for the upper jaw or 0.92 for the lower jaw. This point is supported by the fact that the items are well correlated with their index. These findings strengthen the previous argument that the instruments have a high internal consistency.

Because RD is relatively one of the cheapest prosthodontics treatment modalities for individuals who cannot afford dental implants, the RD is widely used in developing countries [41, 42]. There is a disagreement between the studies regarding the level of patient satisfaction with RDs [34, 43, 44]. Moreover, patient perception and expectations have a noticeable impact on patient satisfaction [45, 46]. For example, denture comfort and mastication ability are critical factors for some individuals, but aesthetics and retention are the essential factors for others [47]. A considerable number of studies proved that many factors impact patient satisfaction. The most common sources of discomfort in the RD patients are bad fit of RPD (33.6%), chewing ability (29.5%), natural tooth problems (26.3%), overall patient perception (26.2%), intraoral cleanliness (20%), speaking (18%), aesthetic (17.8%), and RD cleanliness (15.3%) and unpleasant odor (13.2%) [34, 35]. Some researchers believe that the most common reasons for patients' dissatisfaction with partial dentures are the abutment teeth: condition, number, and position in the arch [48]. Also, the type of denture material, support, and major connectors impact the level of patient satisfaction [48]. Because many variables may impact patient satisfaction, small sample size is insufficient to reach the needed certainty. In other words, a large sample size may be necessary for more precise estimation, generalize the results, and reduce uncertainty. [49, 50]

Different methods and strategies were developed to collect the data. Patient satisfaction could be estimated effectively through one of two forms; interviews and questionnaires [2]. In a structured interview, the interviewer asks the questions in a standard method using one of three approaches: face-to-face, telephone, or computer-assisted personal interviews. The most used approach is a face-to-face interview [2, 51]. In this approach, the interviewer can establish a friendly atmosphere. Therefore, he will be able to improve the participants’ cooperation and raise the level of survey response rates [2, 51]. Above that, he can clarify what the participants may think ambiguous question, resolve any vague participant’s answer, or, if possible, seek more in-depth information or follow-up clarification [2, 3]. On the other hand, the face-to-face interview is relatively expensive and time-consuming. Sometimes, it is not applicable when large sample size is required or when the participants are far from the interviewer. Therefore, telephone interviews or computer-assisted personal interviewing can be considered a better approach [52]. These approaches are less time-consuming and give the researcher the ability to increase the sample size. However, they have limitations and disadvantages. [52] For example, not all the targeted participants can be reached through the telephone or do not have computer or computer knowledge or typing skills. Subsequently, the results produced from the collected data may be considered biased [52]. On the other hand, the valid and reliable self-administered questionnaire can be used directly in the clinic or sent via mail or email to reach more participants. Above that, there is no need to arrange interview appointments, and the participant can fill out the questionnaire at their convenience. Therefore, it can increase the sample size with less bias and lower cost and effort.

Dental literature suffers a lack of valid and reliable instruments in the Arabic language that estimate patient satisfaction with different types of dental treatment, and further studies are needed in this regard.

## Conclusion

The Arabic version of patient satisfaction with the upper RD questionnaire and index and patient satisfaction with the lower RD questionnaire and index are self-administered instruments with good face validity, good construct validity and moderate to excellent reliability. The four instruments can be used in clinical studies to investigate patient satisfaction with any type of RD.

An electronic copy of the German instruments and their translation into English is available under this link: https://qi.quintessenz.de/qi/downloads/qi_alJaghsi_appendix.pdf. An electronic copy of the Arabic version is available in the Additional files 1 and 2.

## Supplementary Information

**Additional file 1**. Patient Satisfaction with Lower Removable Denture Questionnaire, Arabic Version

**Additional file 2**. File 2: Patient Satisfaction with Upper Removable Denture Questionnaire, Arabic Version.

## Data Availability

The datasets used and/or analysed during the current study available from the corresponding author on reasonable request.

## Abbreviations

CD: Complete denture
RD: Removable denture
RPD: Removable partial denture
ICC: Intra-class correlation coefficient
