# Supplementary material for: Validity and reliability of new instruments for measuring patient satisfaction with removable dentures, Arabic Version
Source: BMC Oral Health. 2021 Sep 15;21:446. doi: 10.1186/s12903-021-01811-w (PMC8442818; doi:10.1186/s12903-021-01811-w)
Supplement: Supplementary file 1 — Additional file 1. Patient Satisfaction with Lower Removable Denture Questionnaire, Arabic Version [file 12903_2021_1811_MOESM1_ESM.pdf]

Patient Satisfaction with Lower Removable Denture Questionnaire

Arabic Version

جامعة عجمان
قسم التعويضات السنية

استبيان لتقييم رضا المرضى عن الأجهزة السنية المتحركة في الفك السفلي

عزيزتي المريضة، عزيزي المريض،

جميع المعلومات والأجوبة في هذا الاستبيان سرية، دون أي استثناء.

هذا الاستبيان هو أداة لجمع المعلومات من المرضى حول رضاهم عن أجهزتهم السنية المتحركة في الفك السفلي.

عادة ما يستغرق ملئ الاستبيان مدة لا تزيد عن بضع دقائق.

يرجى الإجابة على الأسئلة بعناية وبشكل كامل ودون مساعدة من أشخاص آخرين.

الاسم: ____________________ التاريخ: ___________

تاريخ الميلاد:___________ الجنس: ذكر ☐ انثى ☐

الجنسية:___________

رقم: _________

يرجى الإجابة بهذه الطريقة	X
رجاء" لا تجب هكذا	•
رجاء" لا تجب هكذا	X

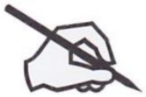

مستوى رضا المريض عن جهازه السني المتحرك في الفك السفلي

	جيد جدا	جيد	ليس بالجيد ولا بالسيئ	سيئ	سيئ جدا
1. ما مدى رضاك عن جهازك السني المتحرك في الفك السفلي **بشكل عام؟**					
2. ما مدى رضاك عن ثبات جهازك السني المتحرك في الفك السفلي؟ **(ضع في الاعتبار حركة الجهاز باتجاه الأعلى)**					
3. ما مدى رضاك عن استقرار جهازك السني المتحرك في الفك السفلي؟ **(ضع في الاعتبار حركة الجهاز الافقية لليمين ولليسار وللأمام وللخلف)**					
4. ما مدى رضاك عن الدعم لجهازك السني المتحرك في الفك السفلي؟ **(ضع في الاعتبار حركة الجهاز عندما تغلق فمك بإحكام)**					
5. إذا اردت ان تقيم أثر الجهاز على **النطق**، ما مدى رضاك عن جهازك السني المتحرك في الفك السفلي؟					
6. إذا اردت ان تقيم أثر الجهاز على **المضغ**، ما مدى رضاك عن جهازك السني المتحرك في الفك السفلي؟					
7. إذا اردت ان تقيم أثر الجهاز على **مظهرك**، ما مدى رضاك عن جهازك السني المتحرك في الفك السفلي؟					
8. إذا اردت ان تقيم **سهولة تنظيف الجهاز**، ما مدى رضاك عن جهازك السني المتحرك في الفك السفلي؟					

شكرا لتعاونكم
